# Reconstruction of Hind and Mid-Foot Defects after Melanoma Resection Using the Reverse Sural Flap: A Case Series

**DOI:** 10.1055/s-0037-1604473

**Published:** 2017-08-03

**Authors:** Juan Jose Larrañaga, Pedro Ignacio Picco, Alejandro Yanzon, Marcelo Figari

**Affiliations:** 1 Department of General Surgery, Reconstructive Surgery Unit, Hospital Italiano de Buenos Aires, Buenos Aires, Argentina; 2 Department of General Surgery, Head and Neck Surgery Unit, Hospital Italiano de Buenos Aires, Buenos Aires, Argentina

**Keywords:** reverse sural flap, foot melanoma, heel reconstruction, sural flap, neurocutaneous flap

## Abstract

**Background** Melanoma resection creates important soft tissues defects, which are difficult to manage when located on the weight-bearing heel and mid foot. There is little evidence on the use of the reverse-flow sural flap for this type of reconstruction.

**Objective** This study reports our case series on the reconstructive management of the hind and mid-foot defects after melanoma resection using the reverse sural artery flap.

**Materials and Methods** This is a retrospective study of four consecutive patients treated with resection of melanoma of the feet and reconstruction with reverse sural artery flap from 2006 to 2009.

**Results** The mean age of the patients was 54 years, three were females, and one was male. Three of the defects were located on the weight-bearing heel, the other on the mid-foot dorsum. The melanomas were fully resected with wide margins. Three patients were reconstructed primarily, whereas one patient was reconstructed 4 weeks after the resective surgery. This series revealed 100% flap survival and there was no partial necrosis. Major complications were not observed. The four patients completely recovered the function of the affected limb.

**Conclusion** The reverse sural flap is a viable option for the reconstruction of foot defects after melanoma resection.

Despite its low incidence, melanoma is the most common malignant neoplasm of the foot and ankle. 1 To achieve local control of melanoma, large surgical margins are required, thus creating important soft tissues defects. Defects located in the weight-bearing heel or in the superior mid foot cannot be reached by conventional local flaps and may be of dimensions not suitable for local flaps. The optimal type of reconstruction should provide sufficient vital tissue with low morbidity and acceptable recovery of the foot function.

There are many reports 2 3 4 5 6 that reveal the utility of the reverse-flow sural flap for the treatment of foot defects originated by traumatic events or diabetic foot and ulcerous disease. Despite many authors have addressed the use of these flaps as a reconstructive option for defects after melanoma excision, no large series have been reported for that purpose. 7 8 9

We support the use of the reverse sural artery flap as the first choice for the management of foot defects derived from melanoma oncologic resection. We present our case series and long-term results.

## Materials and Methods

This is a retrospective study of four consecutive patients on whom resection of melanoma of the feet and reconstruction with reverse sural artery flap was performed at the General Surgery Department of Hospital Italiano of Buenos Aires from 2006 to 2009. Data acquired from patients' medical charts included the primary tumor site, Breslow and Clark histopathologic stage, nodal status, clinical and radiological evidence of hematogenous metastasis, and outcome of surgical treatment ( Table 1 ). 10 11 The surgical indications included patients who had a positive incisional or punch biopsy of the lesion informed as melanoma, with no signs of hematogenous or nodal metastasis on the complete corporal computed tomography scan. These patients had also visited the department of dermatology for preoperative dermatoscopic study, to detect new suspicious nevi. Our multidisciplinary tumor board decided the oncologic management of all patients based on their preoperative oncologic staging and posterior sentinel node status.

**Table 1:** Patient characteristics

Characteristic	Case 1	Case2	Case 3	Case 4
Age (y)	52	44	69	45
Comorbidities	Diabetes	Smoking	Diabetes Hypertension Smoking	Smoking
Location of melanoma	Instep	Heel	Heel	Heel
Breslow (mm)	8	In situ	2.19	5.6
Clark	IV		III	IV
Resection margin (cm)	2	1	2	2
Time of reconstruction	Primary	Secondary	Primary	Primary
Donor-site closure	Primary closure	Primary closure	Secondary closure	Skin graft
Sentinel node biopsy	Positive	Negative	Negative	Negative
Secondary inguinal dissection	Yes	No	No	No
Complications	Cellulitis of the thigh	–	–	–
Follow-up (mo)	74	86	64	60
Recurrence/metastasis	Yes	No	No	No

## Operative Technique

**Fig. 1:**
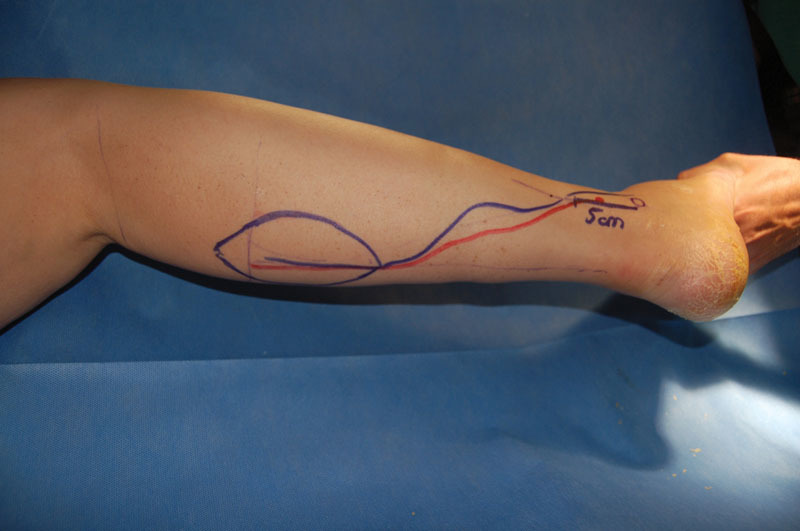
The flap design.

**Fig. 2:**
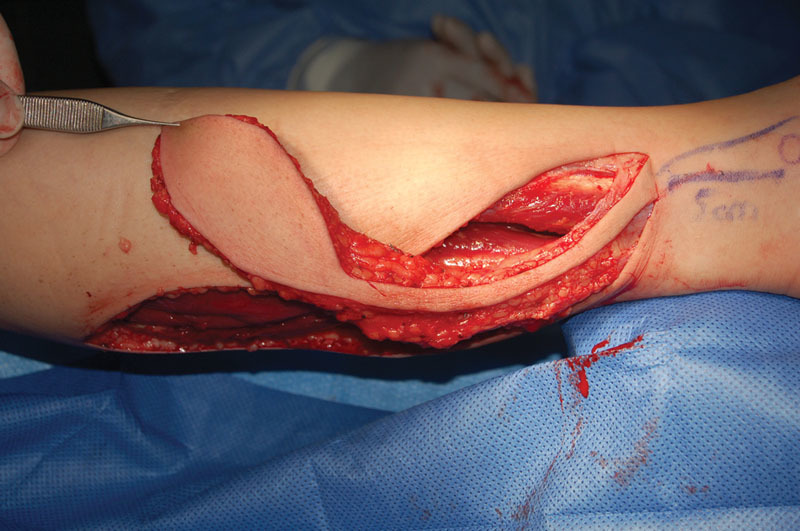
Flap raising (skin covered pedicled).

**Fig. 3:**
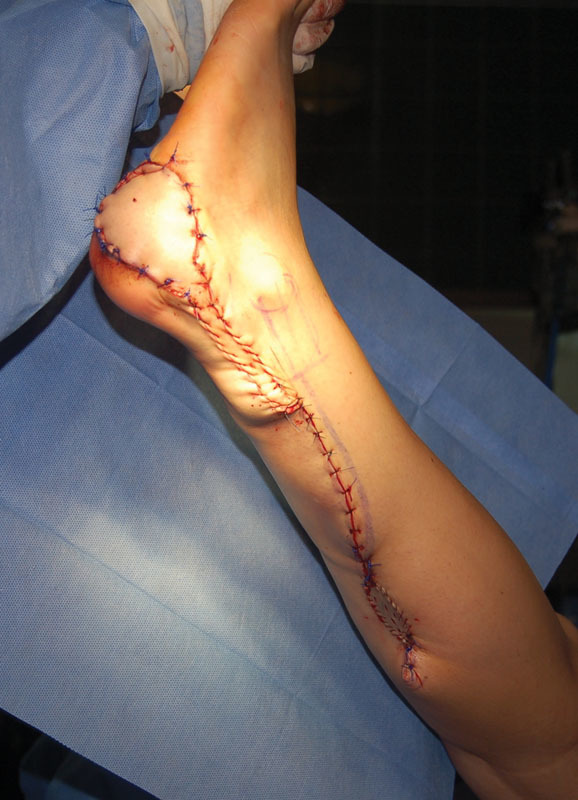
The final position of the flap.

Under general anesthesia, the melanoma was resected with at least 2 cm lateral and deep surgical margins when possible or including the deep fascia when necessary for melanomas of intermediate Breslow. For “in situ” melanomas, a surgical margin of 1 cm was considered as adequate. All flaps were harvested including the medial sural nerve, the lesser saphenous vein, and the deep fascia (see Figs. 1 and 2 ). All the skin paddles were designed and centered at the midline of the calf between the superior and middle thirds. The pivot point of the pedicle was preoperatively marked 5 cm cephalic to the lateral malleolus. Nevertheless, the true pivot point was intraoperatively decided based on the direct identification of a perforating vessel from the peroneal artery. The pedicle was tunneled or rotated including a small strip of skin to prevent compression (see Fig. 3 ).

At the end of the surgery, sentinel lymph node biopsy was performed with the aid of radioactive tracer and patent blue dye.

Patients were encouraged to early mobilization of the affected limb, starting at the first day after surgery. During rest, 20-degree leg elevation was prescribed. No pressure was applied on the flap until the fourth postoperative week. Prophylactic low-molecular-weight heparin was administered to the patients in the immediate postoperative period (enoxaparin 0.4 mg).

## Results

**Fig. 4:**
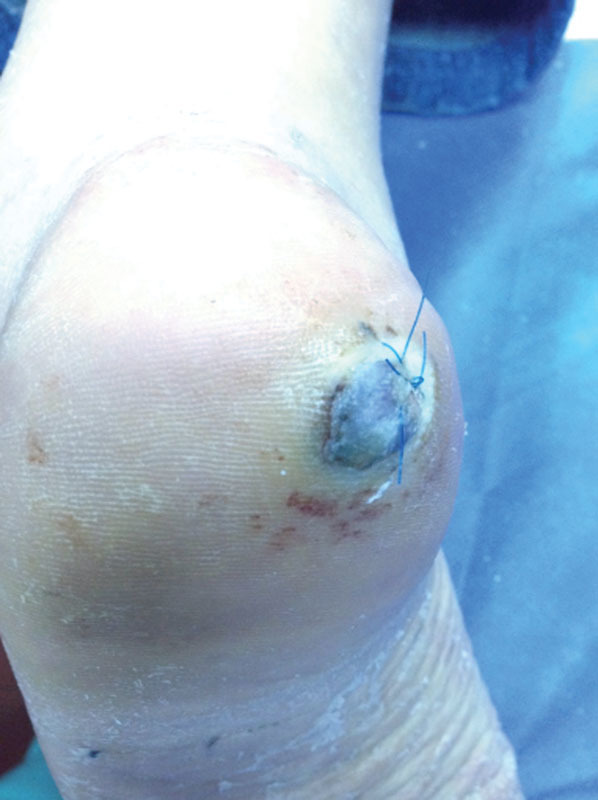
Heel melanoma.

**Fig. 5:**
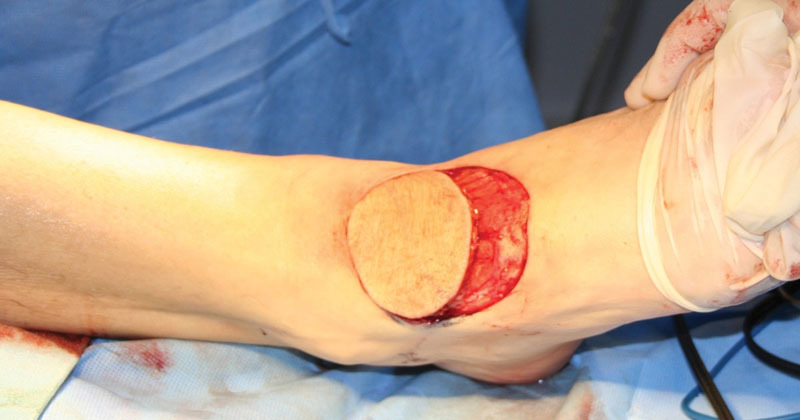
Tunelized flap.

Among the four studied patients, aged between 44 and 69 (mean age: 54) years, three were females, and one was male. Three of the patients presented at least one vascular risk factor, such as diabetes, hypertension, or smoking. Three of the defects were located on the weight-bearing heel, while one was located on the mid-foot dorsum (see Fig. 4 ). The depth of the tumor varied from a melanoma in situ to 8 mm (Breslow [ Table 1 ]). The melanomas were fully resected with appropriate margins. The sural reverse flap was indicated as the first reconstructive option for these patients. The mean size of the skin paddles was 42 cm ^3^ (range: 25–63 cm ^3^ ). All the pedicles were at least 3 cm wide. The pedicle was tunneled in one case (see Fig. 5 ) and in the rest of the cases it was rotated including a small strip of skin to prevent compression. Three patients were reconstructed primarily, whereas one patient was reconstructed 4 weeks after the resective surgery. The mean surgical time was 120 minutes (range: 60–150 minutes). This series revealed 100% flap survival and partial flap necrosis was not observed. Mild venous congestion was noted during the first postoperative week in all cases. Three donor defects were closed primarily and one needed a 2 × 3 cm skin graft. Two patients required a flap debulking procedure for cosmetic and functional reasons. These were performed 4 weeks after the flap transposition in both cases.

**Fig. 6:**
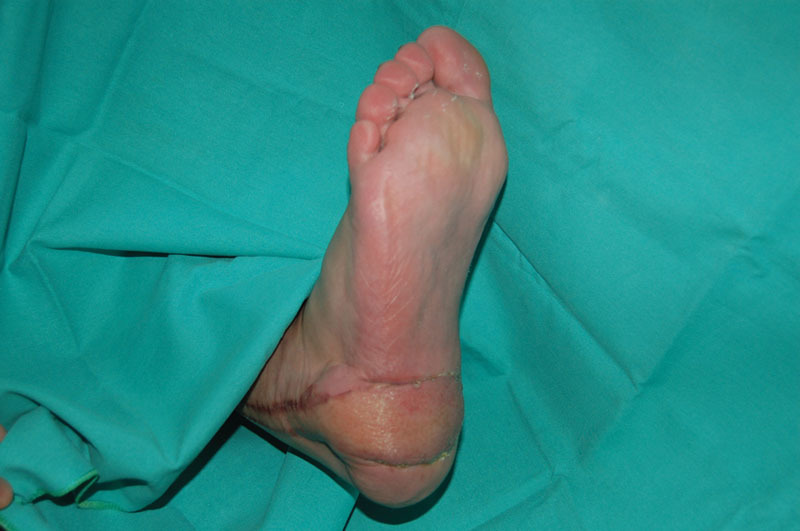
Postoperative appearance after 1 month of surgery.

There were no major complications except for a mild cellulitis of the thigh perceived in patient number one who also had an inguinal lymph node dissection that required oral antibiotics. Cosmetic results were considered acceptable in all cases (see Fig. 6 ). After a minimal 5-year follow-up, the four patients have completely recovered the function of the affected limb, being able to practice any kind of exercise. All the patients have admitted that they experience some numbness in the area of the sural nerve dermatome. However, none of them has considered it as relevant.

## Discussion

The reconstruction of the weight-bearing heel and the mid foot poses a difficult challenge for the reconstructive surgeon. Melanoma resection with oncologic margins generates a considerable impairment of the region; this implies an aesthetic and functional problem difficult to solve mainly because of the lack of acceptable size local flap options. As a matter of fact, flaps obtained from distant locations are needed. Free flaps are an acceptable but complex reconstructive option. 12 Masquelet et al described in 1992 the neurocutaneous reverse sural flap, 13 that offers an efficient method to resurface soft tissue defects around the foot and ankle region with good functional outcomes. It provides sufficient amount of soft tissue and demands less surgical time.

When reviewing the available literature, we can find many authors who recommend the use of the reverse sural flap, 4 6 7 8 9 while others inform poor results. 14 This flap can offer a good amount of viable tissue, the harvesting is very straightforward and fast and the dorsal mid foot and the heel can be reached with little tension. However, many articles report a high incidence of partial flap necrosis. 6 7 8 In addition to that, some authors believe that the reverse sural flap is not suitable for weight-bearing heel reconstruction due to the numbness of the heel induced by the harvest of the flap. This has not been a problem in our patients. Moreover, numbness is not a major concern. Preoperative counseling of the patients on this matter helps the patients to overcome this inconvenience, which finally becomes less uncomfortable in a few months. No lesions have been documented due to this situation.

According to the literature, the reverse sural flap has been widely used for orthopedic or traumatic cases. 3 6 When malignant melanomas involve the foot, large defects are created after the tumor excision with wide margins. Different authors support the use of the reverse sural flap as a reconstructive alternative. 7 8 9

We believe this is a reliable flap for this kind of situation and it offers a less morbid repair. Therefore, free flaps or medial plantar flaps, which leave a large mid-plantar defect to be grafted, are preserved as a second choice.

## Conclusions

The reverse sural flap in this selected group of patients has proven to be an adequate choice for reconstruction with low morbidity and few postoperative complications.

The use of this type of flap in elective and clean situations may be a factor that positively influences the outcome of these procedures, but this should be demonstrated in a larger series.
